# Health professionals’ and coroners’ views on less invasive perinatal and paediatric autopsy: a qualitative study

**DOI:** 10.1136/archdischild-2017-314424

**Published:** 2018-02-08

**Authors:** Celine Lewis, Melissa Hill, Owen J Arthurs, John C Hutchinson, Lyn S Chitty, Neil Sebire

**Affiliations:** 1 North East Thames Regional Genetics Service, Great Ormond Street Hospital, NHS Foundation Trust, London, UK; 2 Genetics and Genomic Medicine, The UCL Great Ormond Street Institute of Child Health, London, UK; 3 Department of Radiology, Great Ormond Street Hospital for Children NHS Foundation Trust, London, UK; 4 Department of Histopathology, Great Ormond Street Hospital For Children NHS Trust, London, UK

**Keywords:** post mortem, autopsy, less invasive, perinatal, paediatric

## Abstract

**Objective:**

To assess health professionals’ and coroners’ attitudes towards non-minimally and minimally invasive autopsy in the perinatal and paediatric setting.

**Methods:**

A qualitative study using semistructured interviews. Data were analysed thematically.

**Results:**

Twenty-five health professionals (including perinatal/paediatric pathologists and anatomical pathology technologists, obstetricians, fetal medicine consultants and bereavement midwives, intensive care consultants and family liaison nurses, a consultant neonatologist and a paediatric radiologist) and four coroners participated. Participants viewed less invasive methods of autopsy as a positive development in prenatal and paediatric care that could increase autopsy rates. Several procedural and psychological benefits were highlighted including improved diagnostic accuracy in some circumstances, potential for faster turnaround times, parental familiarity with imaging and laparoscopic approaches, and benefits to parents and faith groups who object to invasive approaches. Concerns around the limitations of the technology such not reaching the same levels of certainty as full autopsy, unsuitability of imaging in certain circumstances, the potential for missing a diagnosis (or misdiagnosis) and de-skilling the workforce were identified. Finally, a number of implementation issues were raised including skills and training requirements for pathologists and radiologists, access to scanning equipment, required computational infrastructure, need for a multidisciplinary approach to interpret results, cost implications, equity of access and acceptance from health professionals and hospital managers.

**Conclusion:**

Health professionals and coroners viewed less invasive autopsy as a positive development in perinatal and paediatric care. However, to inform implementation a detailed health economic analysis and further exploration of parental views, particularly in different religious groups, are required.

**What is already known on this topic?**

- Autopsy examination remains the gold standard in the investigation of perinatal, infant and child deaths.
- There has been a significant decline in uptake of autopsy globally.
- Less invasive methods involving autopsy involving autopsy imaging techniques with or without laparoscopic-guided tissue sampling have been developed in part to address declining uptake.

**What this study adds?**

- Health professionals and coroners viewed less invasive autopsy as having a number of procedural and psychological benefits over full autopsy.
- Concerns around the limitations of the technology and implementation challenges prior to widespread clinical adoption were identified.
- Formal guidance to ensure application in appropriate settings, detailed economic costing and evaluation of acceptability to patients is required.

## Background

Autopsy (postmortem) examination remains the gold standard in the investigation of perinatal, infant and child deaths adding important clinical information in up to 76% of cases.^1^ Yet uptake rates have declined globally over recent years.^2–6^ In the UK, more than 50% of parents decline postmortem,^7^ a decision many bereaved parents later regret.^8^ As a result of advances in technology and concerns around declining uptake rates, less invasive methods of autopsy have been developed in recent years. Non-invasive autopsy (NIA) uses cross-sectional imaging techniques such as CT or MRI along with ancillary investigations such as microbiology and placental examination. This has the advantage of negating the need for body incisions, while maintaining high concordance with traditional autopsy in the perinatal, paediatric clinical and forensic settings.^9 10^Minimally invasive autopsy (MIA) combines imaging with laparoscopic or image-guided tissue sampling. This approach requires only a small incision thereby reducing the overall cosmetic impact while providing tissue for analysis.^11^

Successful implementation of any new clinical pathway requires thorough evaluation to ensure acceptability among key stakeholders and examination of barriers to uptake, economic evaluation and an assessment of service and policy implications. To date, few studies have been conducted to assess attitudes of parents^12–14^ and health professionals^15^ towards less invasive versus standard autopsy. In a cross-sectional questionnaire to assess acceptability of MIA among healthcare professionals, Ben-Sasi *et al* reported that 40% thought it was more and 50% equally acceptable as full autopsy. As part of an National Institute for Health Research Health Technology Assessment feasibility study regarding acceptability of less invasive autopsy, we report the findings from qualitative interviews conducted with health professionals and HM Coroners to assess acceptability, predicted uptake and issues for service delivery.

## Methods

This was a qualitative study using semistructured interviews and a purposive sampling approach.

### Recruitment

Health professionals across the UK from a range of clinical backgrounds who would be involved in discussions with parents about autopsy examination or would conduct or interpret autopsy results were identified by the authors, purposively sampled and invited via email to participate in the study (table 1). A similar approach was used to recruit HM Coroners. The interviews were conducted by CL either face to face or by telephone between April 2016 and July 2017.

**Table 1:** Participant details

Total participants	29
Profession	
Bereavement midwife	6
Anatomical pathology technologist	4
HM Coroner	4
Intensive care consultant	4
Obstetrics/fetal medicine consultant	4
Perinatal/paediatric pathologist	3
Intensive care unit family liaison nurse	2
Consultant neonatologist	1
Paediatric radiologist	1
Gender	
Female	17
Male	12
Location	
London	16
Regional England	13
Offering/conducting NIA or MIA*	
NIA	13
MIA	6

MIA, minimally invasive autopsy; NIA, non-invasive autopsy.

### Topic guide

**Figure 1:**
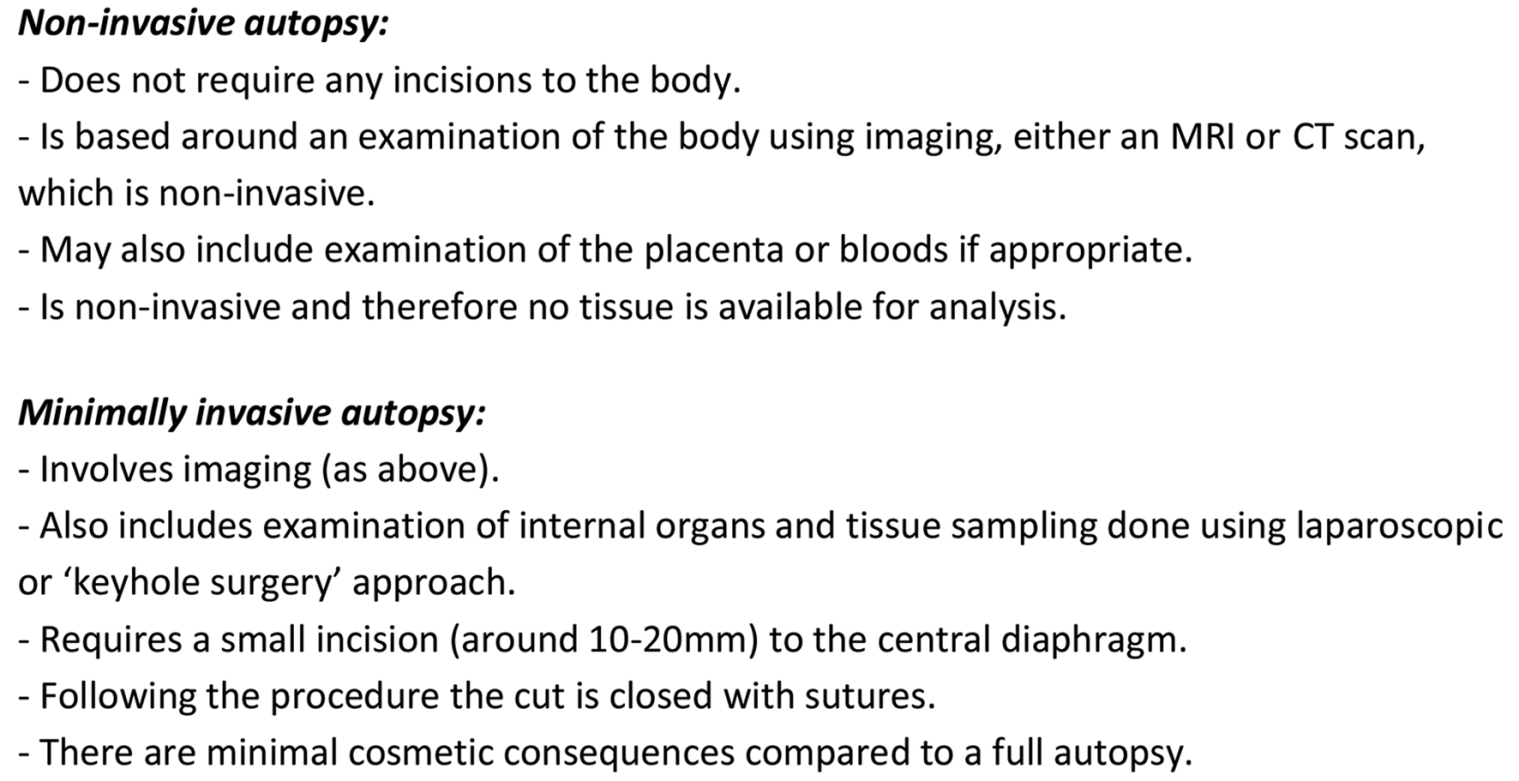
Description of non-invasive autopsy and minimally invasive autopsy given to interview participants. Note: a description of a full autopsy was not provided during the interview as all participants were familiar with the procedure.

The semistructured discussion guide explored participants’ views towards NIA and MIA, perceived benefits, potential limitations or concerns, and implementation into clinical or coronial practice. At the start of the interview, participants were provided with a standardised overview of NIA and MIA (figure 1).

### Analysis

Data were analysed using thematic analysis supported by Nvivo V.10 (QSR International Pty) software.^16^ Data collection and analysis was performed concurrently. The first transcripts were coded independently by CL and MH, and a coding framework was agreed. Subsequent transcripts were coded by CL or MH, and a coding comparison run on Nvivo to ensure inter-rater reliability. Coding disagreements were discussed and resolved. CL coded the remainder of the transcripts according to the framework with subthemes added as appropriate. Final themes were reviewed and agreed by both authors. Data collection continued until saturation was reached.

## Results

### Sample characteristics

Forty health professionals were contacted and 25 took part, from 11 different hospitals (63% recruitment rate) (table 1). Ten coroners were approached: one declined, five did not respond and four took part (40% recruitment rate). Nine health professionals were already offering NIA in clinical service and four coroners explained it was available in their jurisdictions at a cost, although none recalled using it for infant or childhood deaths. Six health professionals were offering MIA either as part of a study or a clinical service; no coroner was offering MIA for coronial cases.

Four overarching themes were identified: (1) benefits, (2) concerns, (3) implementation issues and (4) predicted uptake. These are presented below with illustrate quotes in tables 2–4. Benefits of MIA and NIA are given in table 2.

**Table 2:** Quotes illustrating benefits of less invasive autopsy

Theme	Illustrative quotes
Procedural benefits	
NIA may provide greater diagnostic accuracy	*With sort of very young babies you’re not necessarily going to get as much information as you want with the naked eye, whereas scans are very, very clear.* Anatomical pathology technologist 2
MIA includes benefits of NIA plus tissue analysis	*So I think the imaging is very valuable, however obviously a lot of the times with children we see it’s an infection and you can’t diagnose that from the image.* Anatomical pathology technologist 1
Using images as part of reporting process	*We meet all our families within our service afterwards to discuss the results of the [post mortem]… and I would never show a family here’s a [photograph] of your child’s liver, you know, that would be inappropriate but actually I think I would be comfortable showing them here’s an MRI scan of the brain or the spinal cord…I think actually that would allow you to put a picture on something that you probably couldn’t do now.* Consultant neonatologist 1
Potential to accelerate turnaround times	*I’m assuming it will be quicker because you wouldn’t have to do the lengthy evisceration and evaluation.* Paediatric pathologist 2 *A full PM includes all the other ancillary investigations… to give a final report I have to wait for histology, genetics, microbiology, metabolics. Metabolics can take up to three months.* Paediatric pathologist 3
Psychological benefits	
Procedure more palatable	*When we tell them we’re very clear about what they do in a post mortem and you can see them physically recoil sometimes. I mean we can be as compassionate as we can be whilst we’re trying to explain the details, but it’s often too much for them to take and I really do think a laparoscopic method would be much easier for them to cope with.* Bereavement midwife 3
Familiarity with imaging and laparoscopic approaches	*And often these children have had procedures before where they’ve had you know scans, gastroscopies and surgeries and so they could imagine yes, that’s OK, that wasn’t so bad in the past, that’s something that I could contemplate my child having.* Family liaison nurse 1
Preference for small incisions and biopsies	*I never found that talking about removing tissue samples and putting stuff in wax blocks was an issue. It was just the cutting the head and opening the chest cavity.* Bereavement midwife 2
Choices provide more control for parents	*I think the more choices people have the – it’s so important because they have so little choice. Their child’s died so anything where you can give them some control is really helpful.* Family liaison nurse 1
Benefits to faith groups	
Increased uptake	*There were certainly several Muslim families that I can recall that haven’t been able to agree to a full post mortem, but they happily agreed to just MRI.* Bereavement midwife 1
Positive impact with coroner’s office	*And what’s more, it creates a relationship between the faith communities and the coroner whereby they understand that if you can, you will…So it’s a game changer, it has a hugely positive effect.* Coroner 4

MIA, minimally invasive autopsy; NIA, non-invasive autopsy.

**Table 3:** Quotes illustrating concerns of less invasive autopsy

Theme	Illustrative quotes
Concerns	
Not reaching same level of certainty	*It’s kind of my job to write a report where we really are saying ‘look, we’ve done everything we possibly could’.* Paediatric pathologist 2
Missing or misdiagnosis	*In sepsis or infection actually sometimes the most important thing is the sort of microbiology and sometimes it’s the combination of things which really sort of helps you… you might miss that with a minimally invasive approach.* Paediatric pathologist 1
Inappropriate uptake	*I think a lot of patients would take it up, but I’m worried that some patients would take it up inappropriately and not get the information out of it they’d think they were going to get.* Obstetrician 1
Importance of validation and guidance	*I think you need to make sure the right type of post-mortem is offered to the right type of patient.* Obstetrician 1 *There’s the issue of can you trust the results? And that’s still ongoing.* Coroner 1
Unsuitability of imaging in certain circumstances	*Sepsis, blood infection, you wouldn’t necessarily see any MRI changes, I wouldn’t have thought.* Consultant ICU3 *There’s still a question of whether scans are great for abdominal regions, aneurisms etc.* Coroner 1
Value add of postdeath over predeath imaging	*Is there much mileage in doing a post-death imaging when we’ve already done pre-death imaging? That needs researching, is it something useful? Are you going to see any changes? I don’t know, possibly.* Consultant ICU 1
Tissue required for DNA analysis	*Most of the time what you need is tissue or DNA. And that’s the sort of information on which future pregnancies are advised.* Consultant ICU 4
Potential for false negatives	*I’ve had one recently where there was a tiny cardiac lesion that I didn’t see when I was doing the autopsy, that I only saw on the slides… I’m sort of reflecting saying you would definitely have missed that on a minimally invasive autopsy.* Paediatric pathologist 2
De-skilling workforce	*My concern is that…if we go down that [NIA] route do we deskill our pathologists from doing the full post mortem?… I think we need to consider this aspect. We don’t want to end up being lazy, abandoning the methods that are the best we currently have.* Paediatric pathologist 3 *Will we get a decrease in traditional post mortems because the method is better or because we are getting lazy and when getting consent saying ‘ok, they will consent for a non-invasive, I don’t need to [get] consent for a full post mortem’.* Paediatric Pathologist 3 *We have trainees here that train to be APTs and NIA would kind of take that away from it. I don’t think it would take it completely but in years to come you just don’t know do. Will we be needed anymore?* APT4

APT, anatomical pathology technologist.

**Table 4:** Quotes illustrating implementation issues and likely uptake of less invasive autopsy

Theme	Illustrative quotes
Implementation	
Skills and training	*I’m assuming one of our radiologists would need to have a particular interest in this and would need to be happy to take this on as a particular interest… You need somebody who gets used to looking at them, but whether any of our radiologists have got the will to have an interest in this and to help develop this as a service, that’s something that would need exploring, if it was going to be rolled out.* Obstetrician 1 *I think for some pathologists they’re put off by, you know, trying to use a tiny telescope and just having very minimal access. I think as pathologists we’re used to doing large incisions and dissecting organs… practice changes I suppose this is kind of an extension of that really, just applying it at a post-mortem practice.* Paediatric pathologist 1
Logistics	*I would say that it would be good practice probably for the pathologists and radiologists to look at the images together because the pathologists would have looked at the baby and perhaps may have some other information, might have the clinical history and the radiologist would look at the image…I’d say it’s best done as a joint type of endeavour.* Obstetrician 3 *Personally I think it would be a very, very, very good thing and there’d be lots of advantages but I don’t see it happening for the next ten years unfortunately because of the NHS infrastructure. For example, you need new electronics, broadband, you’ve got to have a wide bandwidth to transmit digital images as well because you might send these images to experts.* Paediatric pathologist 1
Cost implications and equity of access	*It might be something that only happens in five or six centres around the UK.* Radiologist 1 *You need the space to house a scanner. Can you afford it? Can you afford the staff that’s needed? That’s really the block to wide spread development.* Coroner 1 *I would be far, far happier…if it was advertised to everyone, to take away from this being only for faith groups.* Coroner 1
Acceptance and governance	*So I think firstly we need to generate that evidence that MR autopsy is as good and then it would need to gain acceptance within the pathology community and when that has happened I think we would need to do a lot of education of obstetricians and midwives to show that this was a more effective and more acceptable, you know, alternative to a full autopsy.* Obstetrician 3 *I suppose there is always a certain amount of ‘oh, we’ve always done it this way and it works for us’.* Paediatric pathologist 2 *Reconfiguring a service would require a lot of resources and my reflection is it would be a bit of a difficult sell to a management, which I think understandably is very preoccupied about things like the numbers of people coming through the door of A&E [Accident and Emergency] and whether we could get them a bed that night.* Paediatric pathologist 2 *There has been resistance by some Coroners in some areas not least because we haven’t been given much in the way of guidance or information from those who are in a position to understand the science.* Coroner 3
Likely uptake	
	*I think it would be very attractive for parents who don’t want the incision across the head, the Y incision and no, absolutely I think it would be a much larger uptake.* Bereavement midwife 3 *So pretty much people split into ‘do whatever you need to do doc, we need to find out what happened here’ versus ‘you can’t touch my baby’ and there’s just a few in the middle, you know, so you might adjust the margins somewhat.* Consultant ICU 4 *I can certainly think of quite a number of people that you know it was like ‘no, no, no’ no to the full post mortem and then you would say ‘well, actually there is this that we can offer’ and that they would agree to that.* Bereavement midwife 1 *At the moment it’s very much, you know, do you want to the whole autopsy or nothing…So I wonder whether one of the ways that this could change is it wouldn’t be an all or nothing, you could say to parents ‘we will stop at the point at which you tell us and when we get an answer we stop looking’.* Obstetrician 3

### Procedural benefits

Participants acknowledged that there were certain circumstances where imaging would be particularly useful including congenital anatomical abnormalities such as *‘*brain malformations*’*, *‘*cardiac conditions*’* and *‘*skeletal dysplasias*’* and to confirm abnormal prenatal ultrasound findings. Several participants highlighted instances where NIA might provide greater diagnostic accuracy than full autopsy or *‘*show up things you were not expecting*’* such as a variety of abnormalities suggesting a syndrome. The main procedural benefit of MIA was imaging plus tissue sampling increasing the chances of a clinical finding.

Five participants noted the potential benefits of showing MRI or CT images to aid explanation of findings when reporting autopsy results to parents. Seven interviewees speculated as to whether NIA would be quicker than full autopsy, others noted that often the ancillary investigations delay reporting. Finally, some participants speculated as to whether NIA and MIA would be more cost effective; however, there was an acknowledgement that this cost saving may be offset by increased uptake. One participant acknowledged that a *‘*hard economic analysis*’* was required.

#### Psychological benefits

These included health professionals and parents *‘*feeling more comfortable*’* discussing NIA and MIA than a full autopsy and NIA removing the need to open the head which was particularly distressing for parents. Some acknowledged parents might feel more comfortable consenting to imaging and laparoscopic approaches with which they were familiar. Regarding MIA, health professionals commented that a small incision alongside a biopsy was more palatable to parents than a large incision and organ removal. One interviewee commented that increasing choices helps parents feel they have more control.

#### Faith groups

Health professionals acknowledged that less invasive methods of autopsy, particularly NIA, would be preferable to members of the Muslim and Jewish community who traditionally decline autopsy as cutting of the body after death is prohibited, but acknowledged that a fast turnaround time would be required to return the body for burial. Two coroners commented that members of these communities had increasingly requested NIA despite having to cover the costs of the scan. One coroner commented on the positive effect NIA had with the faith community calling it a *‘*game changer*’*. No coroners had requests for NIA from families outside the Muslim and Jewish faiths although it was acknowledged that this was probably because it was not widely known about.

#### Limitations of the technology

One of the main concerns raised related to whether one could reach the same level of certainty with NIA and MIA as a traditional autopsy and the potential for missing a diagnosis or misdiagnosis. Participants also worried about parents consenting to NIA or MIA ‘inappropriately’. A great deal of importance was therefore placed on ensuring further validation and developing official guidance. More specifically, there were various circumstances where participants noted that NIA was unlikely to be suitable including ‘infections’, ‘complex cases where tissue from multiple sites is required’, ‘aneurysms’, ‘stillbirth where the baby dies abruptly’ and coronial cases where there was ‘negligent surgery’ or a ‘suspicious death’.

Intensive care consultants discussed whether there would be value add of doing NIA on a child that had already been scanned, although one suggested that NIA might be useful for a neonate ‘with a constellation of congenital abnormalities’. Two participants commented on the importance of tissue for DNA analysis to provide recurrence risk although with MIA the main concern related to whether there was the potential for *‘*false negatives*’*. One participant commented that for complex cases, tissue may be required from multiple organs, thus requiring a ‘maximum minimally invasive approach’. Finally, one participant noted that we still need a ‘way of sampling the brain’ in a minimally invasive way.

#### De-skilling the workforce

Concerns around *‘*de-skilling*’* pathologists to conduct traditional autopsies and know when a full autopsy is required were raised, particularly in relation to NIA. Two anatomical pathology technologists (APTs) also raised concerns around loss of skills in conducting reconstructions and how their role would fit in with these new technologies. One APT commented that perhaps their role could change to take on some of the laparoscopic work or be trained in using scanning equipment.

#### Skills and training

Some of the most frequently cited issues around NIA and MIA related to the training that would be required of radiologists and pathologists to enable them to set up the service and conduct the procedures. For radiologists it was acknowledged that as well as having an interest in NIA, there would be a ‘learning curve because it is a completely different set of reporting’. A paediatric pathologist commented that some pathologists might be ‘put off… trying to use a tiny telescope*’.* Moreover, the current lack of pathologists working in clinical practice was identified as a potential barrier. Others commented on the need to train staff both to have sufficient understanding of the techniques to be able to consent parents and *‘*to make sure the right type of post mortem is offered to the right type of patient’. One of the pathologists queried whether health professionals might be discouraged from consenting parents for a traditional autopsy because of the availability of NIA and MIA.

#### Logistics

Most participants acknowledged that successful implementation of NIA and MIA would require a multidisciplinary approach with pathologists and radiologists working together. Other key logistical challenges concerned having sufficient access to MRI and CT machines, particularly given that ‘everyone’s priority is for the live patients’ as well as having the computational infrastructure to transmit digital images to experts. Some participants raised concerns around whether you would ‘swamp the pathology department’ if there was an increase in uptake.

#### Cost implications and equity of access

A range of costs associated with implementation were identified, including training pathologists and radiologists as well as covering their time in clinic, the cost of the laparoscopic equipment required for MIA, the potential costs of dedicated pathology department MRI or CT machines to cope with the increase in uptake and the cost of offering an out-of-hours service. Concerns around equity of access and feasibility of offering NIA and MIA to all were frequently raised with concerns around less invasive autopsy becoming a ‘postcode lottery’. Three health professionals commented that a pragmatic solution would be to offer MIA and NIA through specialist centres although a midwife acknowledged that some parents may have concerns about their baby being moved to another hospital. In coronial cases, it was acknowledged that NIA is generally only requested by members of the Muslim and Jewish communities with one coroner noting that it should be advertised to everyone.

#### Acceptance and governance

Acceptance that NIA and MIA were reliable alternatives and the will to change current practice on the part of the paediatric pathology and radiology community were identified as key requirement for successful implementation, although it was also noted by a radiologist that ‘simply the fact that it’s novel and I would need some experience or training to do it…is not a reason not to do it’. The need for buy-in from hospital management to fund training and resources including an out-of-hours service to use scanning equipment was also discussed. Two coroners identified the need for guidance from the Royal College of Pathologists as to when MIA and NIA would be acceptable alternatives to a standard autopsy, with one commenting that current guidance requires ‘a thorough examination’. Two coroners highlighted the ‘political will’ that would be required for authorities to fund ‘a comprehensive out-of-hours service’, although one noted that this might be more likely if an economic evaluation showed NIA to be cost-effective.

### Likely uptake

All participants felt that the availability of NIA and MIA would increase uptake of autopsy although this varied from a ‘much larger uptake’ (bereavement midwife) to ‘you might adjust the margins somewhat’ (consultant ICU). For health professionals already offering NIA or MIA, there had already been an increase in uptake. Participants acknowledged that some parents were still likely to have a preference for a traditional autopsy as it was likely to yield the most information. The vast majority advocated offering MIA and NIA using a tiered approach whereby parents' consent to the most invasive option they would accept but that if a diagnosis was made with a less invasive method, nothing further would be done.

## Discussion

Participants in this study viewed less invasive autopsy as a positive development which was likely to increase uptake as parents would find it more acceptable, particularly those for whom current options are morally or religiously objectionable. Nevertheless, numerous challenges associated with implementation and concerns around the limitations of the technology were raised which will need to be addressed before widespread implementation into clinical practice. This study provides a unique, in-depth insight into the views of health professionals and coroners towards less invasive autopsy. Such insights are crucial given that paediatric and perinatal autopsy examination represent the largest group of consented autopsies.^17^

A number of practical challenges described in this study have previously been identified when considering autopsy imaging in adults.^18 19^ Recommendations from that work included standards of practice and training programmes for pathologist, radiologists and APTs (eg, APTs trained to operate scanners and undertake some minimally invasive procedures), imaging to be performed in any hospital equipped with scanning equipment with images then sent to a centre of expertise for reporting and conducting less invasive autopsy within already established centres of pathology addressing concerns around equity of access.^18^ Such strategies, while realistic, require capital investment and support from clinicians as well as hospital decision-makers, Royal Colleges and local authorities. A detailed costs and benefits economic analysis to determine the true cost of implementing the service is therefore required.

Health professionals identified numerous situations where imaging could be as reliable as or even superior to full autopsy, comments that are supported by the current evidence on NIA.^20–22^ As much of the success of the procedure is highly dependent on the fetus/child being studied, the equipment used and the skills of the reporting team, further evaluation of MIA when offered as a clinical service will be important. Nevertheless, there were some circumstances where it was unclear whether imaging would useful. This highlights the importance of research to understand which circumstances are most suitable for which method of autopsy and development of formal guidance, both to ensure application in appropriate settings and to inform clinicians’ consultations with bereaved parents regarding the likely yield of imaging or other investigations so that informed decisions can be made. As part of this Health Technology Assessment feasibility study, information has been collected regarding the value (or not) of examination and histological sampling of specific organs by clinical indication to help guide parental decision-making.

Further work is also required to determine reporting times for MIA, which may have the benefit of being quicker and improve on the current figure of 60% of autopsy reports meeting the NHS England recommended 42-day turnaround time.^23^ Lengthy reporting times have been identified as a critical issue for parents, many of whom feel they cannot move on until they receive a result.^24^ The need for approaches to reduce laboratory processing time and sample analysis has been identified as key to creating a viable clinical service.^25^ This may apply to NIA, but MIA will still require histological examination.

### Limitations

Participants were self-selecting and there may be responder bias towards people who have strong views towards NIA and MIA. There was a low response rate for coroners (40%). This may be because it was not always possible to contact coroners directly. Finally, this research was only conducted with health professionals and coroners in the UK, opinions outside the UK may differ.

## Conclusion

Health professionals and coroners in this study viewed less invasive methods of autopsy as a positive development in perinatal and paediatric care which could potentially increase uptake. Nevertheless, the practical challenges associated with implementing these technologies will need to be addressed before they can be implemented into routine clinical practice. An economic analysis to determine the true costs and benefits of implementing the service is therefore required as is further research to assess acceptability and likely uptake with parents and religious groups.
